# Epidemiological and molecular characterization of HBV and HCV infections in HIV-1-infected inmate population in Italy: a 2017–2019 multicenter cross-sectional study

**DOI:** 10.1038/s41598-023-41814-x

**Published:** 2023-09-09

**Authors:** Maria Teresa Maggiorella, L. Sernicola, O. Picconi, E. Pizzi, R. Belli, D. Fulgenzi, C. Rovetto, R. Bruni, A. Costantino, S. Taffon, P. Chionne, E. Madonna, G. Pisani, A. Borsetti, C. Falvino, R. Ranieri, R. Baccalini, A. Pansera, F. Castelvedere, S. Babudieri, G. Madeddu, G. Starnini, S. Dell’Isola, P. Cervellini, A. R. Ciccaglione, B. Ensoli, S. Buttò

**Affiliations:** 1 National HIV/AIDS Research Center, Istituto Superiore di Sanità, V.le Regina Elena 299, 00161 Rome, Italy; 2 Core Facilities, Istituto Superiore di Sanità, Rome, Italy; 3 Department of Infectious Diseases, Istituto Superiore di Sanità, Rome, Italy; 4 National Center for Immunobiologicals, Research and Evaluation, Istituto Superiore di Sanità, Rome, Italy; 5 Infectious Diseases Service, Penitentiary Health System, Azienda Socio-Sanitaria Territoriale Santi Paolo e Carlo, Milan, Italy; 6 Cerba Health Care, Milan, Italy; 7 ASST Spedali Civili, Brescia, Italy; 8 Infectious Diseases Unit, Department of Clinical and Experimental Medicine, University of Sassari, Sassari, Italy; 9 Belcolle Hospital, ASL Viterbo, Viterbo, Italy; 10 ASL Roma 4 Civitavecchia, Civitavecchia, Italy

**Keywords:** HIV infections, Hepatitis B, Hepatitis C

## Abstract

HBV/HCV co-infection is common in HIV-1-infected prisoners. To investigate the characteristics of HIV co-infections, and to evaluate the molecular heterogeneity of HIV, HBV and HCV in prisoners, we carried-out a multicenter cross-sectional study, including 65 HIV-1-infected inmates enrolled in 5 Italian detention centers during the period 2017–2019. HIV-1 subtyping showed that 77.1% of inmates were infected with B subtype and 22.9% with non-B subtypes. Italian nationals were all infected with subtype B (93.1%), except two individuals, one infected with the recombinant form CRF72_BF1, and the other with the HIV-1 sub-subtype A6, both previously not identified in inmates of Italian nationality. Non-Italian nationals were infected with subtype B (52.6%), CRFs (36.8%) and sub-subtypes A1 and A3 (5.2%). HIV variants carrying resistance mutations to NRTI, NNRTI, PI and InSTI were found in 7 inmates, 4 of which were never exposed to the relevant classes of drugs associated with these mutations. HBV and/or HCV co-infections markers were found in 49/65 (75.4%) inmates, while 27/65 (41.5%) showed markers of both HBV and HCV coinfection. Further, Italian nationals showed a significant higher presence of HCV markers as compared to non-Italian nationals (*p* = 0.0001). Finally, HCV phylogenetic analysis performed in 18 inmates revealed the presence of HCV subtypes 1a, 3a, 4d (66.6%, 16.7% and 16.7%, respectively). Our data suggest the need to monitor HIV, HBV and HCV infections in prisons in order to prevent spreading of these viruses both in jails and in the general population, and to implement effective public health programs that limit the circulation of different genetic forms as well as of viral variants with mutations conferring resistance to treatment.

## Introduction

The inmate population represents a model of closed community subject to restrictive conditions that has a multinational composition and a high incidence of infections. It is estimated that around 3.8% of the global prison population is living with HIV^1^. Prevalence of HIV infection in jail differs greatly in different regions with proportion greater than 10% in low- and middle-income countries^2^. In Italy, the prevalence of HIV infection among inmates has been estimated to be about 7.5%^3^.

Resident non–Italian nationals are 8.4% of the Italian general population (Italian National Institute of Statistics, 2020). However, according to the December 2022 official data of the Italian Ministry of Justice, non-Italian national inmates in Italian detention centers represented 31.5% of the total inmates. Therefore, non-Italian national inmates are overrepresented in the Italian prisons. Consequently, prevalence of HIV subtypes in prison can be influenced by the presence of non-Italian nationals, regardless of the geographic areas of origin. Our previous data on the prevalence of HIV-1 genetic forms circulating in the immigrant population in Italy demonstrated that almost 80% of them are represented by non-B subtypes and recombinant forms (CRFs)^4^. In contrast, the HIV-1 B subtype was the main genetic form found in the Italian general population, although other subtypes and CRFs have been identified and their prevalence is increasing overtime^5–7^.

Because of the similar modes of transmission of HIV, HBV and HCV, patients with behaviors at risk for HIV infection are also at risk for HBV and HCV infection. HBV/HIV-positive individuals are also at increased risk for developing chronic HBV infection^8^. In addition, HCV may also have an impact on the clinical management of HIV infection^9,10^. In fact, previous studies suggest that HCV co-infection may have an impact on HIV disease progression also in combined antiretroviral therapy (cART) virally suppressed patients^11,12^.

Global prevalence of HBV infection in people living with HIV has been recently estimated to be 7.6%, with the greatest burden in sub-Saharan Africa and in people who inject drugs, with odds of HBV infection 1.4 times higher as compared to HIV-negative people^13^.

Global prevalence of HCV infection in people living with HIV has been estimated to be 2.4% in the general population, increasing up to 82.4% when considering people who inject drugs. Odds of HCV infection are six times higher in HIV-infected people than in their HIV-negative counterparts^14^.

In prison, the prevalence of HBV and HCV infections among the HIV-seropositive inmates is higher than in the general population. This is due to several factors such as poor living conditions in jails, overcrowding, promiscuous intercourses, reduced perception of risk of infection, and absence of an effective health policy^15^, but also to the greater prevalence of injecting drug users^16^.

Worldwide, HBV and HCV co-infections among HIV-people in prison have been estimated to be 12% and 62%, respectively^17^. However, prevalence rates increase to 15% and 78%, for HBV and HCV infections, respectively, among the HIV-positive drug-injecting inmates^17^.

A highly variable prevalence of HIV and viral hepatitis infections has been reported in Italian prisons^18,19^. This varies for HBV and HCV depending on the number of drug users and immigrants present in each detention center. An Italian multicentre study showed, for example, infection of HBV/ HCV and HIV/ HCV, among inmates of 0.4% and 0.9%, respectively^19^. Furthermore, in the last few years HCV treatment with direct acting antiviral (DAA) seems to be achievable even in prison settings, given the short duration of treatment and its high efficacy. This intervention is important to reduce the circulation of HCV and represent an effective prevention strategy^20^.

Regarding HBV infection, the most frequent genotypes in the Italian population are D and A^21^, although other genotypes have been introduced due to immigration, mainly from Africa^22,23^. Concerning the HCV genotype, distribution of variants differs between Italian and non-Italian national populations, genotype 4 is much more prevalent among non-Italian residents whereas genotypes 1b and 2 are the most frequent ones among Italian natives^24^.

The prevalence of HIV, HBV and HCV infections in detention centers raises concerns for their spreading within inmates and to the general population. Thus, although public health interventions in jails have been undertaken to limit spreading of these infections, there are limitations due to the lack of adequate information technology, severe budget constraints, frequent inmate transfers among prisons, absent or poor clinical information-sharing among facilities, and the lack of an effective correctional healthcare database^25,26^.

Health programs may limit the spread of HIV, HBV and HCV infection and the circulation of the different genetic forms of these viruses, as well as of the variants with mutations conferring resistance to treatment. Study of the drug-resistant pattern may, in fact, provide opportunities for improvements of prevention practices and transmission of drug-resistant viruses.

Here we describe a cross-sectional multicenter study to assess the epidemiological, clinical and molecular characteristics of HIV, HBV and HCV infections in HIV-infected inmates during the period 2017–2019, in order to provide date required for targeted public health interventions.

## Results

### HIV infection

Table 1 reports the demographic, clinical, immunological, virological and behavioral characteristics of the study population.

**Table 1:** Demographic, clinical, immunological, virological and behavioral information available for the 65 participants to the study.

Variable	Number (%, unless specified)
**Gender**	
Male	58 (89.2)
Female	7 (10.8)
**Age, median yrs (IQR)**	45 (13)
**Nationality**	
Italy	45 (69.2)
Sub Saharan Africa	6 (9.2)
East Europe	6 (9.2)
North Africa	5 (7.7)
Latin America	3 (4.6)
**cART**	
PI-based	22 (33.8)
InSTI based	15 (23.1)
PI-InSTI based	12 (18.5)
NRTI-NNRTI based	7 (10.8)
Unknown	9 (13.8)
**HIV RNA**	
Aviremic (< 40 RNA copies/ml)	44 (67.7)
Viremic (≥ 40 RNA copies/ml)	9 (20.4)
Unknown	12 (18.5)
**Median Lymphocyte counts (IQR)**	
Total lymphocytes^**a**^	2216 cells/µl (1118 cells/µl)
CD4 + T cells^**b**^	621 cells/µl (476 cells/µl)
CD8 + T cells^**c**^	974 cells/µl (429 cells/µl)
**At-risk behavior**	
Drug abuse and unprotected intercourses	27 (41.5)
Drug abuse	26 (40.0)
Unprotected intercourses	10 (15.4)
Unknown	2 (3.1)

^**a**^On 50 inmates.

^**b**^On 56 inmates.

^**c**^On 54 inmates.

Fifty-eight out of 65 inmates were males (89.2%) and seven females (10.8%). Median age was 45 years with an Interquartile Range (IQR) of 13 years. Forty-five inmates (69.2%) were Italian nationals and 20 (30.8%) were nationals of other countries including 6 inmates (9.2%) from sub-Saharan Africa (Nigeria and Liberia), 6 (9.2%) from East Europe (Albania, Croatia, Macedonia and Romania), 5 (7.7%) from North Africa (Tunisia, Morocco and Egypt), and 3 (4.6%) from Latin-America (Peru and Brazil).

All participants were on cART for HIV but with different treatment regimens. For 9 inmates, details on the drug regimen were not available. Despite the cART, nine inmates (20.4%) were HIV viremic with a range of detectable HIV plasma viral load from 41 to 85,314 copies/ml. Median lymphocyte counts were 2216 cells/µl, 621 cells/µl and 974 cells/µl, for total lymphocytes, CD4^+^-T lymphocytes and CD8^+^ T lymphocytes, respectively.

The declared behaviors at risk for blood-borne virus infections were abuse of drugs (injective and/or non-injective), unprotected heterosexual/homosexual intercourses and tattooing. All inmates reported having unprotected intercourses or being drug addicts or having both the at-risk behaviors; specifically, 41.5% of inmates declared drug abuse together with having unprotected sexual (homo- and/or hetero-sexual) intercourses, whereas 40% reported only the drug abuse, and 15.4% only unprotected intercourses.

**Figure 1:**
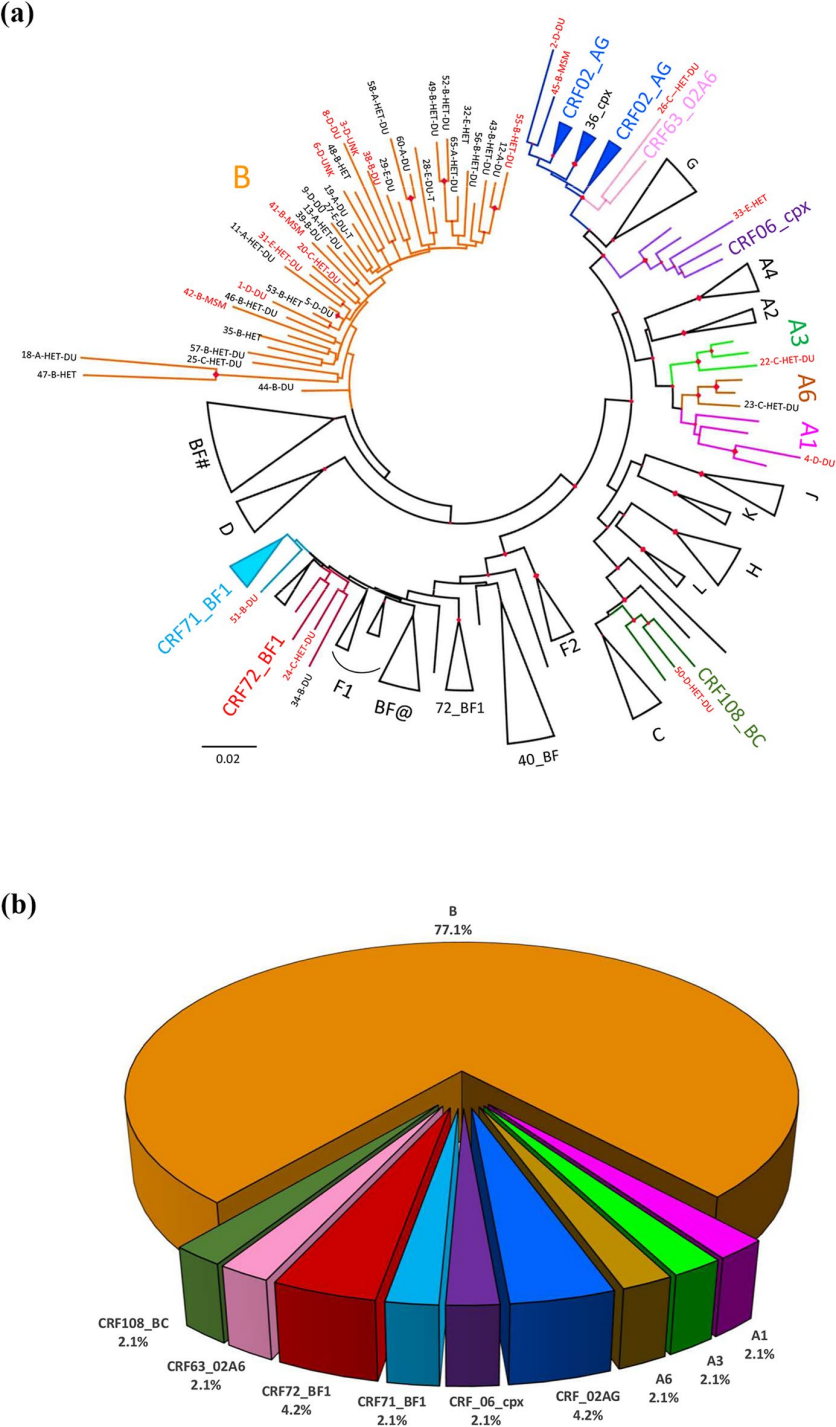
ML phylogenetic tree of HIV RT-PR sequences obtained from 48 HIV-1-infected inmates and genetic forms distribution. Panel a: The phylogenetic tree was inferred by using the Maximum Likelihood (ML) method and the General Time Reversible model^71^. The tree with the highest log likelihood (- 20,708.86) is shown. The analysis involved 138 nucleotide sequences, including 48 infected inmates and 91 sequences from the genome reference set available at https://www.hiv.lanl.gov/ (see Methods for details on the reference set construction). Analyses were conducted in MEGA X^71^. In the tree, clades including sequences from inmates are highlighted in different colours: A1, fuchsia; A3, bright green; A6, brown; B, orange; CRF71_BF1 light blue, CRF72_BF1 red, CRF108_BC green, CRF06_cpx purple, CRF63_02A6 pink, CRF02_AG blue. Clades not including sequences from inmates are represented as cartoons (BF CRFs are indicated with the following labels: BF#: CRF44_BF, CRF38_BF, CRF89_BF, CRF17_BF and CRF12_BF; BF@: CRF40_BF and CRF71_BF). Red labels and black labels indicate sequences from inmates of non-Italian and Italian nationality, respectively. The number indicates the sample code, the first letter is an arbitrary code indicating the detention center, followed by an acronym indicating the risk behavior (HET: unprotected hetero intercourses; MSM: men who have sex with men; DU: drug users; T: tattoo). Diamonds highlight nodes with a bootstrap value > 70%. Panel b: The pie chart shows the distribution of HIV-1 subtypes and recombinant forms in 48 HIV-infected inmates.

To investigate the HIV genetic forms, a phylogenetic analysis was carried out using sequences of the HIV-1 Protease (PR)-Reverse Transcriptase (RT) regions of the *Pol* gene from 48 HIV-infected inmates (Fig. 1, panel A). The Maximum Likelihood (ML) analysis showed that 37 individuals were infected with the HIV-1 B subtype (77.1%), 3 with the sub-subtypes A1, A3 and A6, respectively (2.1% each subtype), and 8 with recombinant forms (16.7%). In particular, 2 inmates were infected with the CRF02_AG (4.1%), 2 with the CRF72_BF1 (4.1%), and 4 with only one among CRF63_02A6, CRF71_BF1, CRF108_BC and CRF06_cpx (2.1% each CRF), respectively (Fig. 1, panel B). Twenty-nine (60.4%) of the 48 characterized inmates were Italian nationals: 27 (93.1%) were infected with B subtype, while the remaining 2 (6.9%) were infected with non-B subtype (A6 and CRF72_BF1). Among the 19 (39.6%) inmates of non-Italian nationality, 10 (52.6%) were infected with HIV-B subtype, 7 (36.8%) with the CRFs, and 2 (10.5%) with the sub-subtypes A1 and A3, respectively.

We also investigated the presence of mutations in the PR, RT and the Integrase (IN) regions of the HIV *pol* gene conferring resistance to HIV drugs (detected according to the 2021 HIV drug resistance database of the Stanford University (https://hivdb.stanford.edu/). Seven out of the 48 subtyped inmates (14.6%) presented Major Drug Resistance mutations (DRMs) (Table 2). In 6 of these we found DRMs in the PR and RT regions, and in one inmate a DRM only the IN region. These DRMs were all found in B-subtypes strains isolated from inmates of Italian nationality, but one that was found in a CRF02_AG strain isolated from a inmate of Nigerian nationality. Table 2 shows the DRMs found in the 7 HIV subtyped inmates, according to the drug class mutation and to the current and previous antiretroviral treatment.

**Table 2:** DRMs in 7 HIV-1 subtyped inmates.

DRMs based on the Stanford University List	Treatment regimens	Therapy drugs
**NRTI-resistance Mutations**		
K70R^	InSTI + NRTI	3TC, ABC, DTG
M184I	InSTI + NRTI	TAF, FTC, EVG, COBI
M184I	PI + InSTI + NRTI	DTG, ABC, 3TC, LPV, TDF, FTC, ATV
M184V	PI + InSTI*	RAL, RTV, DRV
**NNRTI-resistance Mutations**		
K103N	NRTI + PI*	TAF, FTC, DRV, COBI, TDF
**PI-resistance Mutations**		
I84V^	InSTI + NRTI*	3TC, ABC, DTG
I84V	PI + NRTI	FTC, TAF, DRV, COBI, TDF
**InSTI-resistance Mutations**		
R263K	PI + NRTI*	TAF, FTC, DRV, COBI, TDF, ATV, RTV

The abbreviations of the antiretroviral drugs are as follows: atazanavir (ATV), ritonavir (RTV), lamiduvine (3TC), emtricitabine (FTC), tenofovir alafenamide fumarate (TAF), tenofovir disoproxil fumarate (TDF), elvitegravir (EVG), darunavir (DRV), dolutegravir (DTG), raltegravir (RAL), lopinavir (LPV), cobicistat (COBI), abacavir (ABC).

^Mutations in the same sequence.

*Inmates never exposed to relative drug classes.

In particular, K70R and M184I major mutations, associated with Nucleoside Reverse Transcriptase Inhibitors (NRTIs) resistance, were detected in three inmates treated with NRTI -based regimens, whereas the major mutation M184V, associated with NRTI resistance, was detected in an inmate exposed to Protease Inhibitor (PI) + Integrase Strand Transfer Inhibitors (InSTI) and never exposed to NRTIs.

Further, the K103N mutation, associated with Non-Nucleoside Reverse Transcriptase Inhibitors (NNRTI), was detected in an inmate exposed to NRTI + PI and never exposed to NNRTIs.

Finally, the I84V mutation, associated with PI resistance, was present in one inmate treated with PI and in another inmate never exposed to PI. Lastly, the R263K mutation, associated with InSTI resistance, was detected in an inmate exposed to PI + NRTI and never exposed to InSTIs.

Of note, the K70R and I84V mutations were present in the same sequence from a single inmate.

### HBV and HCV coinfections

Inmates were also tested for the presence of HBV and HCV serological markers (Table 3). Four inmates (6.2%) had the HBsAg marker. Twenty-three inmates were positive for the anti-HBs marker (35.4%) and at least another HBV markers with two serological profiles observed (see Table 3), whereas 14 inmates (21.5%) showed the presence of an isolated anti-HBs marker. In agreement with the CDC suggested interpretation of the HBV serologic test results (https://www.cdc.gov/hepatitis/hbv/pdfs/serologicchartv8.pdf), these latter inmates were considered as vaccinated for HBV. Twenty five inmates out of 65 (38.4%) were positive for anti-HBc associated with other HBV markers with two serological profiles observed (see Table 3), whereas 11 inmates out of 65 (17%) showed the presence of an isolated anti-HBc marker. This serological pattern [anti-HBc(+), HBsAg(−) and anti-HBs(−)] often indicates past exposure to HBV with waning anti-HBs immunity, and it is found commonly in HIV-infected individuals, particularly those co-infected with HCV. In fact, 9 of them (81%) were co-infected with HCV (data non shown). No inmates showed the presence of HBV DNA.

**Table 3:** N. of positive inmates/tot. tested for each HBV and HCV infection markers.

HBV infection markers	n/N (%)
HBsAg	4/65 (6.2)
Anti-HBs	
* associated with other markers (total)*	23/65 (35.4)
* anti-HBs* + */HBsAg-/anti-HBc* +	22/65 (33.8)
* anti-HBs* + */HBsAg* + */anti-HBc-*	1/65 (1.5)
* isolated*^a^	14/65 (21.5)
Anti-HBc	
* associated with other markers (total)*	25/65 (38.4)
* anti-HBc* + */HBsAg-/anti-HBs* +	22/65 (33.8)
* anti-HBc* + */HBsAg* + */anti-HBs-*	3/65 (4.6)
* isolated*^b^	11/65 (17.0)
HBV-DNA	0/65 (0.0)

**HCV infection markers**	
Anti-HCV	39/65 (60.0)
HCV RNA	18/65 (27.7)

^a^Isolated anti-HBs: individuals positive for anti-HBs and negative for HBsAg, and Anti-HBc (considered as vaccinated).

^b^Isolated anti-HBc: individual positive for anti-HBc and negative for HBsAg and Anti-HBs.

Regarding HCV serological markers, 39 inmates out of 65 (60.0%) were positive for anti-HCV antibodies, and 18 out of 65 (27.7%) also showed the presence of HCV RNA in plasma. Of these latter, 14 inmates were positive for both HBV and HCV markers, 3 were positive for both isolated anti-HBs and HCV markers and one only for HCV marker, respectively (data not shown). Notably, out of 39 individuals, 13 received anti-HCV DAA-based treatments (33%), 5 spontaneously cleared the virus (13%), 12 had no therapy (31%) and for 9 individuals no data were available (23%) (data not shown).

**Figure 2:**
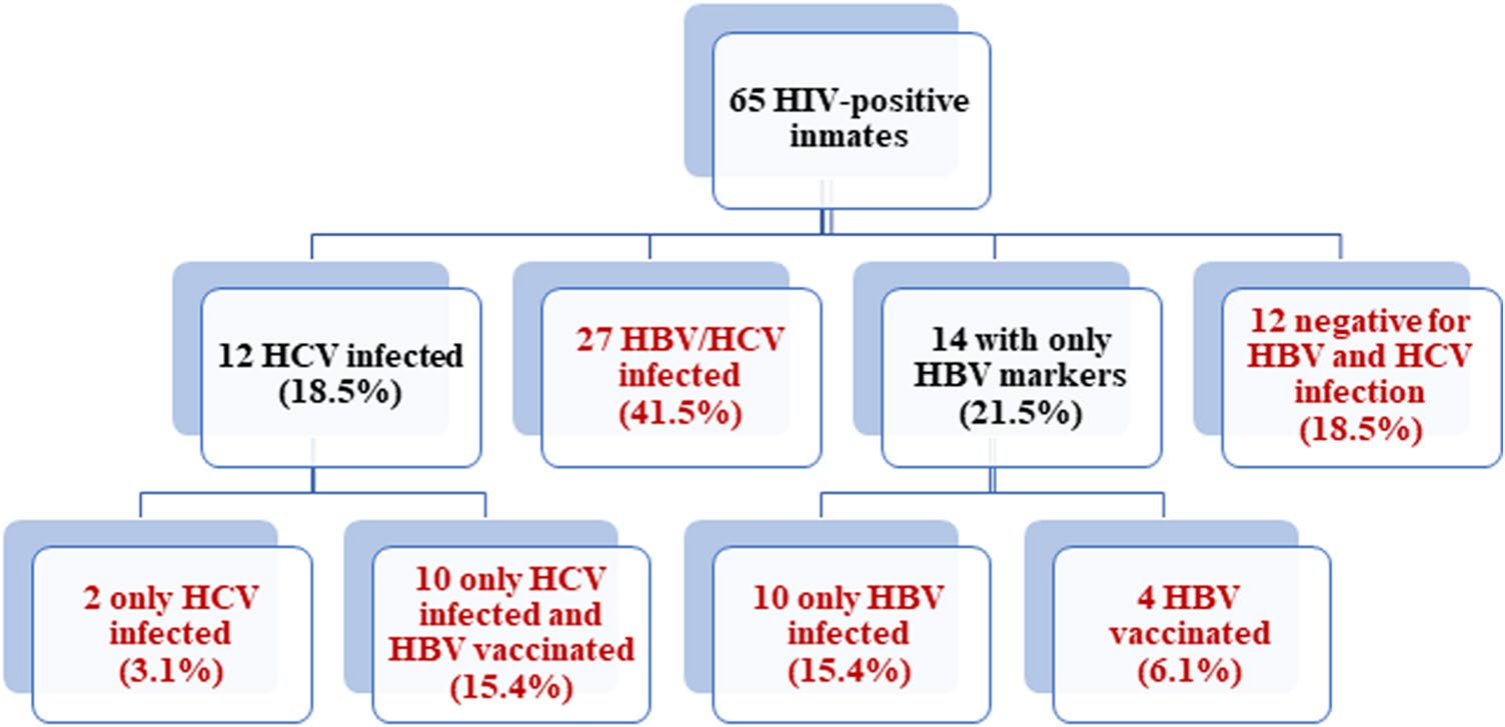
HBV and/or HCV infection and HBV vaccination in 65 HIV-infected inmates. The flow chart shows the prevalence of HBV and HCV infection in 65 HIV-infected inmates. In red the final estimates for HBV and/or HCV infection are indicated.

Figure 2 shows the distribution of HBV and/or HCV coinfections and HBV vaccination in the HIV-infected inmates. In detail, 14 inmates (21.5%) showed only HBV markers; of these, 10 (15.4%,) showed HBV infection markers, while the others 4 (6.2%) were vaccinated for HBV. Among the 12 inmates (18.5%) who showed the presence of only HCV infection markers, 2 (3.1%) were co-infected by HCV, while 10 (15.4%) were HCV co-infected and HBV vaccinated. Twenty-seven (41.5%) were HBV/HCV doubly co-infected (previous or current infection). Finally, 12 individuals (18.5%) were negative for all HBV and HCV markers tested, and had no evidence of current or past co-infection.

The association of the presence of HBV or HCV infection markers with demographic variables was then investigated (Table 4). HBV markers were not significantly associated with either age or nationality of inmates, while the percentage of inmates positive for HCV markers was higher in older than in younger inmates (74.2% vs 44.4%, respectively, *p*-value = 0.0209). The presence of HCV marker was also more frequently associated with the Italian nationality (75.6% in Italian nationals vs 25.0% in non-Italian nationals, *p*-value = 0.0001).

**Table 4:** Prevalence of HBV and HCV markers and their distribution by demographic data.

	HBV markers (% individuals )*			
	N. of inmates	Negative	Positive	*p*-value**
**Age (yrs)**				
23–44	19	36.8	63.2	0.1870
45–65	26	19.2	80.8	
**Nationality**				
Italian	32	18.7	81.3	0.0708
Non-Italian	19	42.1	57.9	

	HCV marker (% individuals)			
	N. of inmates	Negative	Positive	*p*-value
**Age (yrs)**				
23–44	27	55.6	44.4	0.0209
45–65	31	25.8	74.2	
**Nationality**				
Italian	45	24.4	75.6	0.0001
Non Italian	20	75.0	25.0	

*Excluded 14 inmates vaccinated for HBV, 13 from Italy and 1 from Perù.

**Chi Square test.

In order to evaluate determinants associated with the risk of having at least one co-infection, univariate and multivariate ordered logistic models were performed. In the univariate model, non- Italian nationals appeared to be at more risk of having co-infections, whereas higher CD8 + T cell counts, the elderly and the drug abusers, whether or not combined with unprotected intercourses, had a higher risk of being infected with HIV alone. The multivariate model confirmed the CD8 + T-cell counts, age and drug abuse effects, while nationality seemed not to be a true risk factor for having co-infections (Table 5).

**Table 5:** Univariate and multivariate ordered logistic model for the risk of having only HIV, 1 coinfection or 2 coinfections.

	Univariate model			Multivariate model		
	Point estimate	95% Wald		Point estimate	95% Wald	
		Confidence limits			Confidence limits	
**CD8 + T cell counts**	0.999	0.998	1.000	0.999	0.998	1.000
**Non-Italian nationals vs Italian nationals**	2.974	1.092	8.097	3.486	0.834	14.577
**Age (45–65) vs Age (23–44)**	0.335	0.124	0.902	0.243	0.074	0.801
**Drug abuse vs only unprotected intercourses**	0.172	0.045	0.666	0.164	0.036	0.756

In the multivariate model all the variables are each other adjusted.

**Figure 3:**
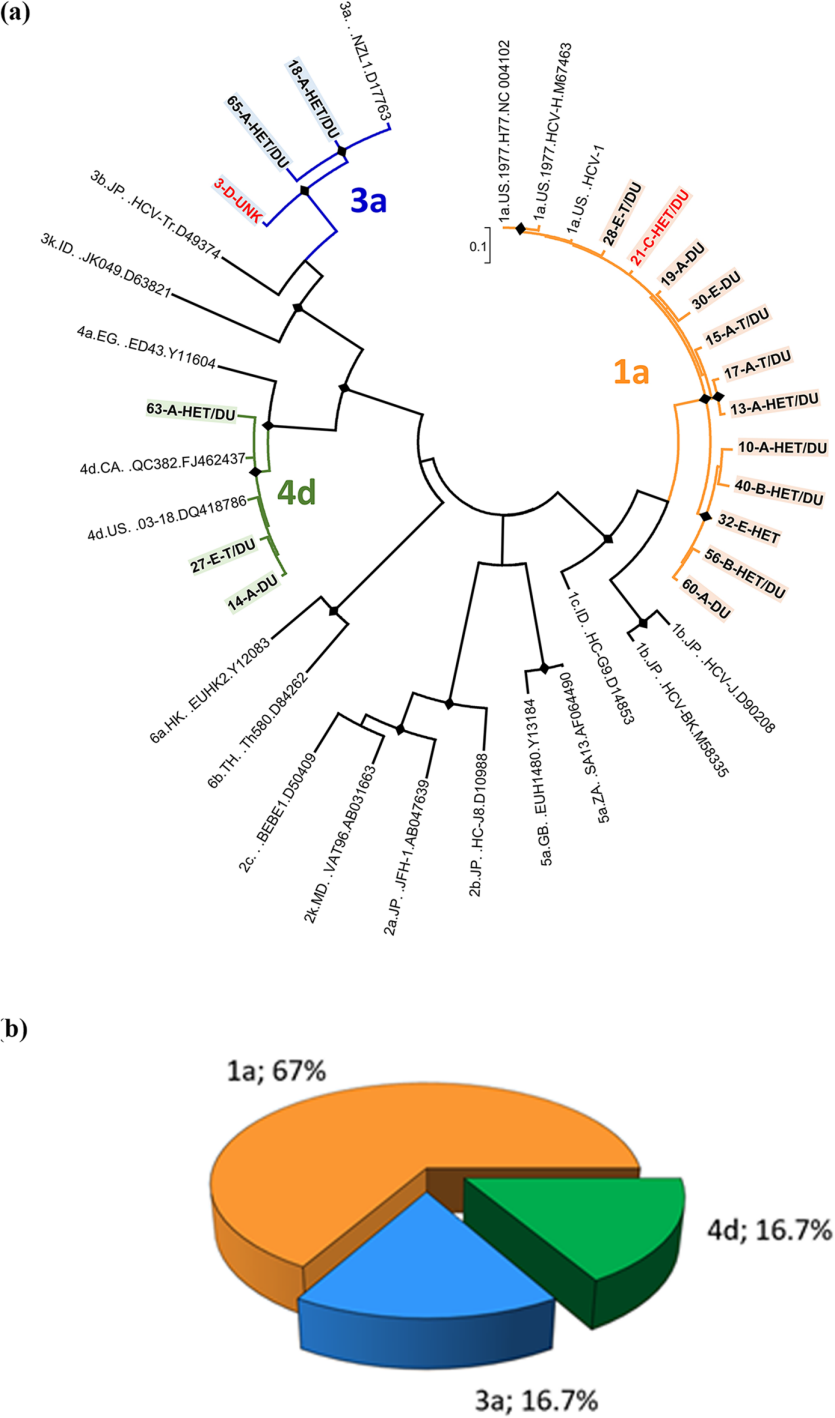
ML phylogenetic tree of HCV NS5B sequences obtained from 18 HCV infected inmates and genetic forms distribution. The tree shows the phylogenetic relationships among the 18 HCV sequences from inmates and 20 reference sequences, representing the major known HCV genotypes/subtypes, downloaded from Los Alamos HCV (https://hcv.lanl.gov/content/index). The best substitution model (T92 + G + I) for the dataset under analysis was preliminarily assessed by the Models function in MEGA. Panel a: subtypes including sequences from inmates are highlighted in different colours: 1a, orange; 3a, blue; 4d, green. The number indicates the sample code, the first letter is an arbitrary code indicating the detention center, followed by an acronym indicating the risk behavior (HET: unprotected hetero intercourses; MSM: men who have sex with men; DU: drug users; T: tattoo). Sequence labels from inmates of non-Italian nationality are reported in red, those from inmates of Italian nationality in black. Diamonds highlight nodes with a bootstrap value > 70%. A black diamond (♦) marks statistically supported nodes (bootstrap value > 70%). Panel b: prevalence of HCV NS5B subtypes in 65 HIV-infected inmates. The prevalence of the genetic forms is expressed as the percentage of the total number.

Figure 3 reports the phylogenetic tree inferred from HCV NS5B sequences. Sequences were obtained from 18 individuals showing the HCV RNA marker in plasma (Fig. 3, panel a). Sixteen of these were Italian nationals. The subtype 1a was by far the most frequent since it was detected in 12/18 inmates (66.6%); subtype 3a and subtype 4d were equally detected in 3/18 inmates (16.7%) (Fig. 3, panel b). The two inmates of non-Italian nationality were from Morocco and Macedonia and were infected by subtype 1a and 3a strains, respectively. Analysis of risk factors referred by the 18 HCV infected inmates showed that all but two were drug users. However, 12 of them also reported unprotected sex.

All samples were negative for HBV DNA.

## Discussion

Our study proposes to analyze the inmate population that represents a model of closed community subject to restrictive conditions and characterized by a multinational composition and a high incidence of disease. In prison, emerging problems such as drug use, HIV/AIDS and other infectious diseases are often present and they must be addressed in both Italian and non-Italian national inmates.

For an accurate surveillance, in prison the monitoring of viral epidemiological changes and circulation of virus strains are pivotal for both risk groups and the general population.

In our study, 30.8% of the enrolled inmates were of non-Italian origin. This data is in line with the 2018 official data (the average time period of sample collection) from the Ministry of Justice, which indicated a 33.9% of inmates of non-Italian nationality (https://www.giustizia.it/giustizia/it/mg_1_14_1.page?facetNode_1=1_5_40&contentId=SST165666&previsiousPage=mg_1_14).

It is well known that HIV-1 subtype B is predominant in Western Europe, including Italy, North America and North Africa^27,28^. However, in recent years, the frequency of non-B clade forms has been reported to increase in subtype B-restricted geographical areas and in Italy^6,29^.

The HIV strains phylogenetic analysis showed that the majority of inmates of Italian nationality were infected with the HIV-1 subtype B, except two, one infected with the recombinant form CRF72_BF1, and the other one with the HIV-1 sub-subtype A6, both previously not identified in inmates of Italian nationality^30,31^. CRF72_BF1 was isolated in Brazil^32^, and it has spread in Spain. HIV-1 sub-subtype A6 is present in the Former Soviet Union (FSU) countries likely originated from A1 strains of African origin, and is now spreading into non-FSU countries^33^. The presence of new strains within a closed community, such as a prison, can become a public health problem because this would allow the spread of new drug-resistant variants.

Conversely, a different pattern was found in the non-Italian inmate population. Ten out of 19 inmates of non-Italian nationality, were infected by subtype B strains. They came from countries where HIV-1 subtype B circulates with the exception of Liberia and Nigeria in which only very few cases of subtype B have been described in the recent past [Los Alamos https://www.hiv.lanl.gov/components/sequence/HIV/geo/geo.html accessed on march 7, 2023; ^29,34^]. Of the other non-Italian inmates, six were infected with subtypes and CRFs circulating in their countries of origin, and three were infected with CRF71_BF1^35^, CRF72_BF1^32^ and CRF108_BC^36^ respectively. Of note, these CRFs were isolated from inmates coming from Tunisia, Romania and Albany, where circulation of these three recombinant forms has not been previously described.

Despite considerable success in the treatment of HIV-1 infection, there continues to be a growing concern about the emergence of HIV-1 drug resistance mutations (DRMs) that can compromise the effectiveness of antiretroviral drugs. It has been described earlier that the prevalence of DRMs in cART naive individuals varies from 0 to 15% and may depend on several variables such as geographical area, HIV prevalence, transmission route and virus subtype^37–40^. However, it must be kept in mind that various factors, such as early treatment, use of appropriate drug regimens, and good adherence to therapy play a major role in blocking the emergence of DRMs^41^.

Since DRMs do not appear during ART in patients with suppressed viremia^42^, HIV-infected ART-naive individuals are the major source of drug-resistant viruses in both the developed and low- and middle-income countries^43,44^. Consequently, the DRMs, that can persist for a significant period of time even in the absence of drug treatment, can be transmitted (TDRMs)^45^. DRMs can be selected during antiviral treatment and persist in proviral DNA as integrated forms in viral reservoirs. Consequently, if treatment is interrupted or adherence compromised, there is a risk that the reactivated virus is drug resistant, potentially facilitating transmission to co-infection partners and hampering the effectiveness of antiviral treatments^45^.

In our study, we found DRMs in 7 HIV-1 subtyped inmates (6 with subtypes B isolated from inmates of Italian nationality, and 1 with CRF02_AG isolated from a inmate of Nigerian nationality). Six inmates had major DRMs in the PR and RT genes, and one inmate had major DRM in the IN gene. Of note, two mutations, K70R and I84V, were present in the same sequence from a single inmate. The simultaneous presence of multiple DRMs within the same viral genome can lead to a significant increase in resistance to antiviral drugs, as well as cross-resistance to inhibitors of the same class^46^. Three out of 6 inmates showed resistance mutations to drugs belonging to classes never administered to them. For one inmate the previous therapeutic regimen was unknown, therefore it cannot be excluded that the DRM could be related to the previous therapy. One inmate, treated with PIs and with InSTI showed a resistance mutation to NRTI drugs. It has been recently described that transmitted mutations to NRTI drugs in patients treated with InSTI can increase the risk of viral failure if treated with InSTI-based regimens^47^. We also found an inmate with the R263K mutation against InSTIs, a class of drugs never administered to him, which confers low-level resistance to InSTIs^48,49^. Other authors have also described a very low prevalence of transmitted drug resistance to InSTIs^50–52^. However, it has been observed that increased resistance rates is associated with incomplete adherence to therapy and low CD4 T-cells regardless of which INSTI was administered^53^. Thus, baseline resistance to INSTIs should be evaluated. In the case of emergence of DRMs to one class of drugs the retreatment with an antiviral of the same class could cause poor response, suggesting that switching to another combination type/class of drugs is preferable^46^. However, it must be appreciated that there are resistance mutations that may appear in patients receiving drugs that affect regions other than those in which the mutations were found. As in the case of DDA resistance for HCV infection, we can define these extra target mutations^46^. This suggests that DRM studies are important when deciding to treat HIV-infected inmates.

We found that 75.4% of HIV-infected inmates showed also markers of HBV and/or HCV infection. In particular, 56.9% of individual had infection markers for HBV, 60.0% for HCV and 41.5% + for both HBV and HCV co-infection. Of note, 14 inmates (13 Italian nationals and 1 Peruvian national, 21.5%) were found positive only for anti-HBs antibodies. Therefore, although we did not have information on the HBV vaccination status of the 65 inmates, we considered these inmates as vaccinated for HBV, as suggested by the CDC (https://www.cdc.gov/hepatitis/hbv/pdfs/serologicchartv8.pdf). Although the Ministerial Decree n. 251 of 25 October 1991 specifies that vaccination against hepatitis B must be administered to all prisoners, this is severely hampered by the high turnover of inmates in Italian prisons. For this reason, rapid vaccination schedules have been proposed and are being applied^54,55^. Stasi and colleagues found a similar prevalence of inmates (15.9%) with isolated anti-HBs marker in detention facilities in the Tuscany region^55^, and considered them as vaccinated.

We found that HBV infected inmates were mostly Italian nationals (81.3% vs 57.9% of non-Italian nationals). This highlights the importance of screening for HBV in accelerating vaccination in order to ensure short-term protection in the prison population^55^.

In our study, 11 (17%) inmates showed an isolated anti-HBc marker. This prevalence is in line with the frequency of anti-HBc marker in HIV-1-infected patients observed by Chang and colleagues. This frequency varied from 17 to 40%, representing, in most of cases, a waning host immune response to HBV infection that can result in HBV reactivation with varying consequences in morbidity^56^. Nine out of 11 inmates in our study showed an isolated anti-HBc pattern associated to HCV marker. The isolated anti-HBc pattern is particularly relevant in HIV/HCV co-infected patients and, most often, HCV is dominant, suppressing HBV replication^57^. When HCV is treated, its inhibitory effects on HBV replication is released possibly resulting in HBV reactivation. In fact, HBV reactivation has emerged in the era of DAA treatment of HCV infection. Due to this, screening for HBV should be performed also in prison prior to initiation of HCV therapy.

In our study, HBV sequencing was not attempted since all samples were found to be negative for HBV DNA. This was expected because HIV treatment includes inhibitors of reverse transcriptase that are also effective against the reverse transcriptase activity of the HBV polymerase.

Our data confirm the results of other studies that showed a high prevalence of HIV/HCV coinfection in inmates. HCV co-infection was significantly associated with the Italian nationality. In Italy, a high prevalence of HCV infection has been reported in drug users, both in the general and in the inmate populations^58–60^. In fact, we found a higher prevalence of this coinfection in inmates older than 45 years (74.2%), corresponding to inmates born in the years at major risk for drug abuse, one of the main transmission routes of HIV/HCV. The multivariate model showed that higher CD8 + T-cell counts, elderly age and drug abuse were associated with HIV infection alone, while nationality seemed not to be a risk factor for having co-infections. In agreement with our data, Schmidt et al.^61^ reported CD8 T-cell failure in HCV and HBV infections, due to T-cell exhaustion, deletion, and viral escape.

The prevalence of HBV and HCV infection markers in HIV-infected prisoners, although high, is lower than what we found in our previous study^31^. The greatest difference was observed considering the HCV infection that was present in 60.0% of inmates in this study, as compared to the 78.3% of the previous study. Similarly, 27.7% of inmates had HCV RNA, a much lower prevalence compared to the 65.2% found in our previous work. It must be specified that inmates enrolled in our previous work were not under anti-HCV DAA-based treatments^31^. Therefore, even if the detention centers involved in our two studies are different, we can speculate that the lower prevalence of HCV markers can be, at least partially, due to administration of DAAs against HCV, as observed in our present study and as already reported by others^62,63^. A recent study indicates that the prevalence of HCV markers among inmates is less than 20%, possibly due to the availability of DAAs^64^. However, other factors may also play a role, such as the improving strategies for the control of infectious diseases, and/or a different distribution of behaviors at-risk for HCV infection among the detention centers. More tailored studies are needed to evaluate the efficacy of DAAs drugs in reducing HCV infection prevalence in prison.

HCV genotype and subtype heterogeneity may be influenced by the route of infection and coinfection, regardless of the geographic variable^65,66^. In this work we found that the subtype 1a is the most frequent variant (66% prevalence), followed by subtypes 3a and 4d that are equally represented with a prevalence around 16.7%. In our previous study^31^, we found a similar HCV subtype distribution with the exception of the prevalence of HCV subtype 3a which was higher than in the present study. In addition, in both studies, we found a similar prevalence of the 4d subtype. This distribution of HCV subtypes is in line with the distribution observed in the population of drug addicts outside the prisons, both in Italy and in Europe^58,67^. However, in the general population, subtype 1b is predominant with a prevalence of more than 50%, followed by genotype 2 (35%), and by subtypes 1a and 3a that show a very low prevalence^68^.

Some limitations of our study should be addressed. Firstly, our study was based on voluntary enrollment and, therefore, we were unable to obtain an estimate of the percentage of HIV-positive people who were enrolled in the study, as compared to the total HIV-positive people in the 5 detention facilities. However, the prisons participating in the study were uniformly distributed in all the Italian territory.

Another limitation of our study is that HIV sequences were obtained from proviral DNA since the majority of the enrolled inmates had negative plasma viral load. In fact, the proviral DNA sequence can contain a variety of multiple DRMs, not present in plasma viral RNA^69^, which may thus reveal viral variants with mutations that are not actively expressed^70^. However, HIV DNA sequencing may provide a good method to gain unique preliminary insights into HIV‐1 subtype diversity or for an overall evaluation of HIV DRM in a population^70^. Data from previous studies have also shown that the proviral compartment can be reliably used for the investigation of DRMs in ART-naive patients^71^.

Finally in our study, we utilized Sanger sequencing as the standard HIV genotyping method. However, this methodology may not detect low–abundance HIV variants mutations, that are less represented within the viral pool. Although Next Generation Sequencing (NGS) is a more sensitive method capable of identifying minor resistant mutants within viral populations^72,73^, a high concordance exists between Sanger sequencing and NGS. Nevertheless, further investigation are needed for detection of low‐abundance HIV‐1 variants by NGS.

In conclusion, our data indicate the need for a systematic screening and monitoring both at the beginning of incarceration and during incarceration to limit the spread of HIV, HCV, and HBV.

Although public health interventions are underway, effective prevention programs should be implemented that can limit the spread and circulation of the different genetic forms of these viruses, in prisons and to the general population, as well as of variants with mutations that confer resistance to treatment.

## Materials and methods

### Study population

A multicenter study was performed from 2017 to 2019 in detention centers of 5 Italian cities (from North to South: Brescia, Milan, Civitavecchia, Viterbo, Sassari). The study was approved by the ISS ethical committees [prot. PRE-866/16 (November 8th, 2016)] and the local ethical committees [Viterbo, Prot. n. 769/CE Lazio1 (March 31st, 2017); Sassari, Prot. n. 2495/CE (May 23rd, 2017); Brescia, Prot. n. 2699 (May 26th, 2017); Civitavecchia, Prot. n. 2287/CE Lazio 1 (November 30th, 2018), Milan, Prot. n. ST-173, 2018 (November 17th, 2018)]. All participants gave written informed consent in accordance with the Declaration of Helsinki. Demographic, behavioral, clinical, immunological and virological data were collected for each patient in complete anonymity in each center in a case report form provided by the National HIV/AIDS Research Center (CNAIDS) of the Istituto Superiore di Sanità (ISS). Sixty-five inmates already diagnosed with HIV infection accepted to participate to the study. After signature of the informed consent, 2 ml of plasma from whole blood, collected in EDTA during the routine testing, were obtained, using standardized procedures in each clinical center. Plasma samples and blood components were kept at-80 °C in each center until shipment to CNAIDS (ISS) in Rome for serological HBV and HCV analyses and sequencing of HIV, HBV and HCV.

### Serological assays

Anti-HCV, anti-HBc (IgM and IgG) and HBsAg markers were determined by chemiluminescent assays on an automated analyzer (COBAS Elecsys e401, Roche Diagnostics, Basel, Switzerland), using Elecsys anti-HCV II, Elecsys anti-HBc and Elecsys HBsAg II kits, the last one with a sensitivity of 20–30 mUI/ml. Anti-HBs antibodies were detected by Enzygnost Anti-HBs II (Siemens Healthcare Diagnostics Products, Germany) and results evaluated according to the manufacturer's instructions.

### Viral nucleic acid quantification

HIV viral load was determined at each center where enrollment was performed, as a common routine analysis for HIV-infected inmates, using different commercial kits.

HBV DNA quantification was performed at ISS on plasma using the High Pure System Viral Nucleic Acid Kit (Roche Diagnostics, Basel, Switzerland), followed by amplification and detection on a COBAS AmpliPrep/COBAS TaqMan Instrument using the COBAS Taq Screen MPX Test v2.0; this assay is currently used for blood screening and has a sensitivity of 2.1 IU/ml.

HCV RNA quantification was performed on plasma using the High Pure System Viral Nucleic Acid Kit (Roche Diagnostics, Basel, Switzerland), at ISS, followed by amplification and detection on a COBAS TaqMan 48 Analyzer using the COBAS TaqMan HCV Test v2.0; this test has a sensitivity of about 9.3 IU/ml and a linear range from 25 to 3.91 × 10^8^ IU/ml.

### Virus genotyping

According to data reported by each center, a few inmates had a detectable HIV viral load, most of them being under successful cART. HIV subtyping was therefore carried out on HIV proviral DNA. To this purpose, HIV proviral DNA was extracted from 400 ul of whole blood using the QIAamp DNA blood mini kit (Qiagen, Hilden, Germany) according to the manufacturer’s instructions. A portion of the *pol* gene, encompassing the Protease and Reverse Transcriptase-encoding region (the PR-RT region) was amplified by a nested Polymerase Chain Reaction (PCR), following a previously described protocol^74^, with modifications. Briefly, primers for PR-RT region amplification were for PCR outer: NES3 5’GAC AGG CTA ATT TTT TAG GG 3’ located at 2075–2094 (gag); NS4 5’ GGC TCT TGA TAA ATT TGA TAT GT 3’ located at 3561–3583 (pol),; PCR inner FK1 5’ AGC AGA CCA GAG CCA ACA GC 3’(2140–2159 gag), FK7 5’ CTA TTA AGT CTT TTG ATG GGT CA 3’ (3506–3528 pol) (with reference to the HIV-1 HXB2 strain from the Los Alamos National Laboratory database using the Sequence Locator and QuickAlign tools: http://www.hiv.lanl.gov/content/ sequence/HIV/mainpage.html).

The region of the *pol* gene encompassing the Integrase-coding region (nucleotides 4150 to 5263 according to the HIV-1 HXB2 sequence) was also amplified. To this purpose, primers for the nested PCR amplification were the following: PCR outer FS1:5’-CATGGGTACCAGCACACAAAG-3’FS6: 5’-CTGTATGCAGACCCCAATATG-3’; PCR inner FS2:5’-GGAGGAAATGAACAAGTAGA-3’ FS5: 5’-GGGATGTGTACTTCTGAACT-3’. The optimized thermocycler conditions for outer and inner PCR assays were: initial denaturation at 94 °C for 2′, 37 cycles at 94 °C for 1′, 55 °C for 1′, 72 °C for 2′ and a final extension at 72 °C for 2′^75^.

The resulting amplicons were purified using Gel Extraction Kit (Qiagen, Hilden, Germany) and directly sequenced using an ABI 3730 automated sequencer.

For HBV, no patient showed the presence of HBV DNA (HBV DNA below the limit of detection of the test), therefore amplification of HBV genome sequences was not attempted.

For HCV, viral RNA was extracted from 140 μl of serum samples using the QIAamp Viral RNA Extraction Kit (Qiagen, Hilden, Germany), according to the manufacturer’s instructions. Healthy donor serum samples were used as a negative control. The RNA was reverse transcribed using the High-Capacity cDNA Reverse Transcription Kit protocol (Thermo Fischer Scientific, Waltham, Massachussetts, US) and the resulting cDNA amplified by nested PCR using the FastStart High Fidelity PCR system (Roche Diagnostics, Basel, Switzerland). The specific primers used to amplify the NS5B region (nt 8256–8632) of HCV for the first and second rounds have been previously described^76^. PCR conditions for both rounds were 94 °C for 2’, followed by 28 cycles of denaturation at 94 °C for 15″, annealing at 60 °C for 30″, extension at 72 °C for 45″, and a last extension step at 72 °C for 7′. PCR products were analyzed on a 2% agarose gel stained with GelRed (Biotium). Both strands were sequenced using the Genome Lab DTCS Quick Start KiT (Beckman Coulter, Inc., Fullerton, CA). Purified sequencing reactions were run on an automated DNA sequencer (Beckman Coulter, Inc., Fullerton, CA).

### HIV and HCV phylogenetic analyses

To perform HIV phylogenetic analyses, a genome reference set was constructed starting from the reference sequences available at the HIV Los Alamos database (www.hiv.lanl.gov) that includes 428 genomes. The sequences were aligned by Clustal Omega (https://www.ebi.ac.uk/Tools/msa/clustalo/).

and a redundancy removal procedure was performed. On the basis of the identity matrix for each pair of sequences with a percentage of identities (%ID) higher than 95%, only one sequence was considered. This resulted in a set of genome sequences including 46 pure subtypes and sub-subtypes (A1-A6 12; B 5; C 3; D 4; F1-F2 7; G 4; H 4; K 2; J 3; L 2) and 186 CRFs. These sequences were aligned with the 48 PR-RT sequences obtained from 48 inmates by Clustal Omega. The resulting alignment were manually edited by using BioEdit 7.2^77^ and gaps were removed. A phylogenetic analysis was carried out by MEGA X by using the Maximum Likelihood (ML) method and the General Time Reversible (GTR) model^78^, the statistical robustness and reliability of the inferred tree was estimated by a bootstrap test (1000 bootstrap replications), and it was visually inspected to remove CRF sequences having no phylogenetic relationships with our 48 sequences. The resulting final reference set comprises 91 sequences (including 46 pure subtypes and sub-subtypes and 45 CRFs). The phylogenetic inference procedure (ML method; GTR model; 1000 bootstrap replications) was repeated on the novel set of sequences to assign inmate sequences to subtypes.

The HCV genome reference set, representing the major known HCV genotypes/subtypes, includes 20 reference sequences downloaded from the HCV Los Alamos databases ((www.hcv.lanl.gov). These sequences were aligned to 18 inmates sequences by ClustalW in BioEdit 7.2^77^, the resulting multiple alignment was manually edited and gaps were removed. Genotype assignments were achieved by constructing a phylogenetic tree inferred using a ML method by MEGA X, using the best substitution model describing the observed data, preliminarily determined by the Models function in MEGA X. The statistical robustness and the reliability of the phylogenetic tree were confirmed by bootstrap analysis using 1000 replicates.

The resulting phylogenetic trees for HCV and HIV data sets were visualized by Fig Tree 1.4.4 (http://tree.bio.ed.ac.uk).

### Determination of HIV Major Drug Resistance Mutations (DRMs) against HIV ART

The presence of major DRMs against HIV ART in the *protease*, *RT* and *integrase* genes was investigated in all inmates, for whom either the PR-RT or the Integrase region nucleotide sequences, or both, were known, according to the WHO-2009 list of resistance mutations^79^, the 2019 IAS-USA Drug resistance mutations in HIV-1 list^80^, and the Stanford HIV Drug Resistance database (Stanford University, version 9.0, 2021; https://hivdb.stanford.edu/hivdb/by-mutations/)].

### Nucleotide sequence accession numbers

The nucleotide sequences of the HIV-1 protease and reverse transcriptase (PR-RT) regions and of the HCV NS5B region obtained from all subtyped inmates are available in GenBank, with the following accession numbers ON611640-ON611686 (HIV-1 PR-RT) and ON055295-ON055312 (HCV NS5B).

### Statistical analysis

Descriptive statistics summarizing quantitative variables included median and interquartile range (IQR). Qualitative variables were presented as absolute and relative frequencies. Two-tailed Pearson’s chi-squared test was used to evaluate the difference in the prevalence of viral hepatitis markers between groups based on demographic. *p*-values less than 0.05 were considered statistically significant.

Univariate and multivariate ordered logistic models were performed in order to evaluate determinants associated to the risk of having 1 or 2 coinfections vs only HIV. In the multivariate model all the variables are each other adjusted.

All the statistical procedures were performed using the SAS®, Version 9.4 (SAS Institute Inc. 100 SAS Campus Drive Cary, NC, USA).

### Ethical approval

The study was approved by the ISS ethical committees [prot. PRE-866/16 (November 8th, 2016)] and the local ethical committees [Viterbo, Prot. n. 769/CE Lazio1 (March 31st, 2017); Sassari, Prot. n. 2495/CE (May 23rd, 2017); Brescia, Prot. n. 2699 (May 26th, 2017); Civitavecchia, Prot. n. 2287/CE Lazio 1 (November 30th, 2018), Milan, Prot. n. ST-173, 2018 (November 17th, 2018)]. All participants gave written informed consent in accordance with the Declaration of Helsinki.

## Data Availability

The datasets generated and analysed during the current study are available in the NCBI repository (https://www.ncbi.nlm.nih.gov/genbank/; Accession numbers: ON611640-ON611686 for HIV-1 PR-RT sequences and ON055295-ON055312 for HCV NS5B).
